# Unsupervised learning of metabolic fingerprints from 3D magnetic resonance spectroscopic imaging enables glioma subtype classification

**DOI:** 10.1093/noajnl/vdaf220

**Published:** 2025-10-23

**Authors:** Gulnur S Ungan, Paul J Weiser, Jorg Dietrich, Daniel Cahill, Ovidiu C Andronesi

**Affiliations:** Athinoula A. Martinos Center for Biomedical Imaging, Department of Radiology, Massachusetts General Hospital, Boston, Harvard Medical School, Boston; Athinoula A. Martinos Center for Biomedical Imaging, Department of Radiology, Massachusetts General Hospital, Boston, Harvard Medical School, Boston; Computational Imaging Research Lab—Department of Biomedical Imaging and Image-guided Therapy, Medical University of Vienna, Vienna, Austria; Department of Neurology, Massachusetts General Hospital, Boston, Harvard Medical School, Boston; Department of Neurosurgery, Massachusetts General Hospital, Boston, Harvard Medical School, Boston; Athinoula A. Martinos Center for Biomedical Imaging, Department of Radiology, Massachusetts General Hospital, Boston, Harvard Medical School, Boston

**Keywords:** IDH-mutant astrocytoma and oligodendroglioma, glioblastoma, magnetic resonance spectroscopic imaging, non-negative matrix underapproximation, unsupervised machine learning

## Abstract

**Background:**

Accurate classification of glioma subtypes is essential for personalized treatment, yet current diagnostic approaches rely on invasive procedures to determine molecular profiles. This study aims to enhance non-invasive glioma classification by integrating metabolic imaging with advanced unsupervised learning.

**Methods:**

Whole-brain 3D Magnetic Resonance Spectroscopic Imaging (MRSI) was performed at 3 Tesla. From 26 scanned patients, 12 gliomas (5 astrocytomas, 5 oligodendrogliomas, 2 glioblastomas) that passed strict quality-control criteria were included for analysis. Spectral decomposition was performed using Global Non-Negative Matrix Underapproximation (G-NMU), and tumor subtype classification was achieved with Uniform Manifold Approximation and Projection (UMAP) followed by K-means clustering.

**Results:**

The proposed framework was able to classify tumor types with an accuracy of 99.65% and an AUC of 99.07. Clear subtype-specific metabolic fingerprints were validated by hierarchical clustering and UMAP embeddings, emphasizing 2HG, serine, and inositol as important classification drivers.

**Conclusions:**

This study demonstrates that whole-brain MRSI spectral decomposition based on G-NMU is a reliable non-invasive method for classifying gliomas. In contrast to spectral fitting on prior-knowledge basis sets, G-NMU accurately separates astrocytoma, oligodendroglioma, and glioblastoma by extracting metabolic features without making assumptions about the tumor metabolic composition. These results suggest that integration of metabolic imaging and unsupervised learning into clinical workflows may improve molecular stratification for noninvasive glioma diagnosis.

Key Points

- This study is the first to apply Global Non-Negative Matrix Underapproximation (G-NMU) to whole-brain 3D MRSI for glioma classification, uncovering distinct metabolic fingerprints that capture both composite neuronal-glial patterns and selective drivers such as serine and inositol.
- Integration of G-NMU with UMAP and K-means clustering achieved near-perfect classification of astrocytoma, oligodendroglioma, and glioblastoma (accuracy = 99.65%, AUC = 0.9907), highlighting the clinical potential of unsupervised metabolic imaging for non-invasive glioma stratification.
- In contrast to prior-knowledge basis set fitting, G-NMU extracts biologically interpretable tumor signatures without assumptions, providing a robust non-invasive framework for glioma diagnosis and stratification.

Importance of the Study

In order to extract metabolic fingerprints without making assumptions about the tumor content, this study presents Global Non-Negative Matrix Underapproximation (G-NMU) for whole-brain 3D MRSI. In contrast to traditional methods, G-NMU makes use of the entire spectrum and reliably distinguishes between glioblastoma, oligodendroglioma, and astrocytoma according to their inherent metabolic differences. This framework finds biologically significant drivers like serine and inositol and achieves near-perfect classification by combining spectral decomposition with unsupervised learning (UMAP and K-means). By improving molecular stratification, these results show that unsupervised metabolic imaging can offer a potent, non-invasive substitute for biopsy. The method provides a scalable framework based on unsupervised decomposition techniques of metabolic imaging that could be applied in neuro-oncology to help with clinical needs, including, diagnosis, treatment planning, and treatment response assessment.

The molecular classification of gliomas has significantly evolved with the revised WHO Classification of Tumors of the Central Nervous System,^1^^,^^2^ emphasizing the molecular biology of tumors over traditional histopathological grading. Molecular markers, particularly IDH mutation status and 1p/19q codeletion, have proven crucial for glioma classification.^1^^,^^2^ While distinguishing IDH-wildtype glioblastoma (wt-IDH) from IDH-mutant gliomas (mIDH) is fundamental for diagnosis and prognosis, an equally critical task is to differentiate between two main mIDH subtypes astrocytoma (AC) and oligodendroglioma (OG). This distinction directly informs patient management, with treatment strategies that diverge substantially between wt-IDH and mIDH, as well as between AC and OG despite their shared mIDH background gliomas.^3-5^

These advances have underscored the need for non-invasive, imaging-based^1^^,^^6^ approaches capable of preoperatively classifying gliomas with molecular precision. While conventional MRI provides valuable information on tumor localization, morphology, and grade, it lacks direct molecular characterization. MR Spectroscopy (MRS) has emerged as a promising tool for interrogating glioma metabolism, offering insights into tumor-specific metabolic alterations.^7-13^ Notably, de-novo 2-hydroxyglutarate (2HG) production by IDH-mutant gliomas and increased cystathionine levels in 1p/19q-codeleted oligodendroglioma can be detected using MRS,^11^^,^^14-20^ enabling non-invasive molecular classification.

Current MRS techniques rely on spectral fitting^21-25^ to separate 2HG and cystathionine from overlapping metabolites, which is challenging for non-edited spectra. However, edited MRS is slower, has lower signal-to-noise and is performed at lower spatial resolution compared to non-edited MRS. Furthermore, spectral fitting assumes a fixed number of known metabolites. This assumption is likely to be violated in cancer, where metabolic alterations can result in completely new metabolites that are not part of prior-knowledge basis sets. In fact, the prime examples of this are exactly 2HG and cystathionine, which prior to the last decade were not part of any basis set and were discarded from the spectral fitting despite a 40-year history of MRS applications for studying glioma metabolism. Hence, analysis of non-edited MRS data that does not depend on spectral fitting and its limiting assumptions to distinguish between metabolic profiles of astrocytoma and oligodendroglioma would be highly useful. Alternative methods, such as Independent Component Analysis (ICA), Non-Negative Matrix Factorization (NMF), and Convex NMF (C-NMF), use advanced feature extraction techniques to capture comprehensive and biologically meaningful metabolic signatures without spectral fitting and are well suited for classification problems.^26-29^ In particular, recent advancements in Non-Negative Matrix Underapproximation (NMU)^30^ have introduced new possibilities for improving MRS-based glioma classification. NMU has shown superior performance over traditional factorization methods, particularly in capturing localized spectral features relevant to tumor metabolism.^31^

This study employs Global Non-negative Matrix Underapproximation (G-NMU), a constrained matrix factorization method that extracts latent metabolic sources and their spatial contributions by ensuring that the reconstructed signal does not exceed the original spectroscopic input at any point. This underapproximation constraint improves interpretability and sparsity, making G-NMU particularly suited for metabolic decomposition in tumor tissue. In addition, unsupervised machine learning techniques, including UMAP for dimensionality reduction and K-means clustering for subtype classification, are integrated into the analysis pipeline to enhance differentiation between glioma subtypes. By combining MR spectroscopic imaging (MRSI), spectral decomposition, and unsupervised learning, this study aims to improve preoperative classification of wt-IDH vs. mIDH, addressing critical gaps in current metabolic imaging approaches and supporting precision neuro-oncology.

## Materials and methods

### Patients

For this study, 26 glioma patients had whole-brain MRSI scans. These patients had tumors that were both high- and low-grade and had different types of cells and molecules. All participants gave their written consent after reading and agreeing to an IRB-approved protocol. The complete cohort comprised 13 astrocytomas, 10 oligodendrogliomas, and 3 glioblastomas. Eleven tumors were rated as Grade 2, eight as Grade 3, and seven as Grade 4 by the WHO. The treatment history showed that 10 patients had never had treatment before, 2 had surgery only, and the other 14 had multimodal treatment, such as surgical resection, radiotherapy, and combinations of chemotherapy.

For this study the 12 patients that were not treated with radiotherapy and chemotherapy were selected for tumor type classification and diagnosis. This subset comprised 5 astrocytomas (mIDH), 5 oligodendrogliomas (mIDH), and 2 glioblastomas (wt-IDH). Five tumors were WHO grade 2, four were grade 3, and three were grade 4.

The age range of the analyzed cohort spanned 25 to 66 years, with a mean age of 39.7 ± 12.1 years and a median of 34 years. Sex distribution was slightly female-predominant, with 4 male and 8 female patients. When stratified by tumor subtype, astrocytoma patients had a mean age of 33.6 years (± 7.5 years, median = 33), oligodendroglioma patients 42.0 years (±12.8 years, median = 34), and glioblastoma patients 57.5 years (±4.94 years, median = 57.5).

All oligodendrogliomas harbored both IDH mutations and 1p/19q codeletion, consistent with WHO 2021 criteria. Astrocytomas exhibited molecular alterations in ATRX, TP53, MGMT, and PI3K. Among the 10 AC+OG patients, eight were treatment-naïve, and two (P5 and P10) were imaged after surgical resection only, allowing interrogation of intrinsic metabolic features of untreated tumors (Table 1).

**Table 1.** Patient demographics, tumor grade, molecular markers (MM), and metabolic index with healthy voxel counts

ID	Age/Sex	Grade	Type	Molecular markers	Tumor	Healthy
P1	33F	2	AC	mIDH, TP53	63	227
P2	34F	3	AC	mIDH	81	246
P3	25M	3	AC	mIDH, ATRX, TP53+. TERT-	26	79
P4	44M	4	AC	mIDH, PI3K, TP53	63	266
P5	32F	2	AC	mIDH	5	143
P6	52M	3	OG	mIDH, 1p/19q	27	91
P7	34F	2	OG	mIDH, 1p/19q	9	166
P8	66F	3	OG	mIDH, 1p/19q	4	32
P9	28F	2	OG	mIDH, 1p/19q	14	78
P10	30F	2	OG	mIDH, 1p/19q	6	23
P11	61M	4	GBM	wt-IDH	18	154
P12	54F	4	GBM	wt-IDH	10	140

### Imaging

Whole-brain 3D MRSI data were acquired using 3 Tesla MRI scanners (Tim Trio and Prisma, Siemens Medical Solutions) equipped with a 32-channel head coil. Data acquisition employed a navigated adiabatic spin-echo spiral-encoded sequence^32^ with a repetition time of 1800 ms, an echo time of 97 ms, a field of view of 220 × 220 × 120 mm³, and an isotropic resolution of 7.3 mm. The spectral acquisition bandwidth was set to 1.2 kHz, and 3 averages were acquired with a total scan duration of 8:12 min: sec. Spectral data were fitted using LCModel software (version 6.3-1R, Provencher), and metabolic maps were reconstructed at 2.4 mm resolution with a feature-based super-resolution pipeline, enhancing spatial precision and metabolite quantification. Further details of the acquisition and processing protocols were provided in Ref. 32.

### Analysis

Strict quality control measures were applied to ensure spectral reliability, selecting the voxels with a full width at half maximum (FWHM) less than 0.15 ppm, a signal-to-noise ratio (SNR) larger than 3, and Cramer-Rao lower bounds (CRLB) smaller than 20% as calculated by the LCModel spectral fitting software. Spectral artifacts were defined by a threshold where the baseline signal had to surpass the mean (μ) by at least 2.5 times the standard deviation (σ). Tumor regions were identified. Tumor regions were identified selecting only voxels where the mIDH metabolic index defined as HGI = [2-Hydroxyglutarate + Glutamine]/[Glutamate] was greater than 1.5 and wt-IDH metabolic index defined as CNR =[total Choline]/[total NAA] was greater than 0.4.

**Figure 1.**
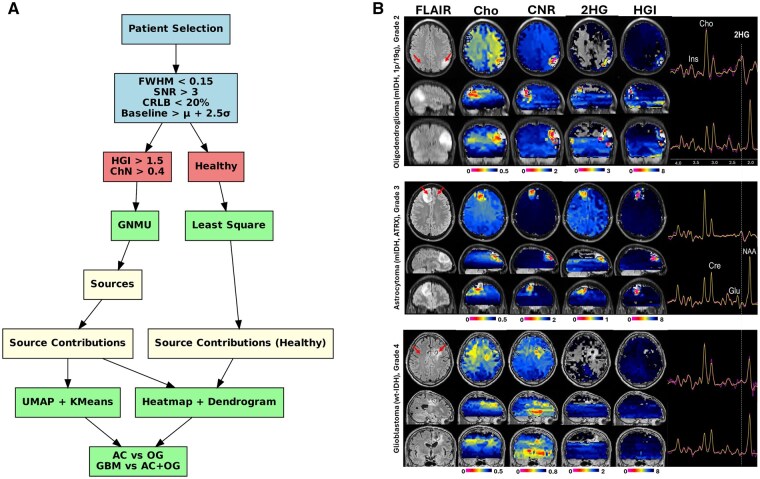
(A) Analysis pipeline for glioma classification using MRSI and NMU. (B) Metabolic maps of AC (top), OG (middle) and GBM (bottom).

Figure 1A shows the flowchart of the study, while Figure 1B shows examples of metabolic maps for astrocytoma, oligodendroglioma, and glioblastoma. G-NMU was applied for the decomposition of selected spectra. G-NMU is a constrained matrix factorization method that decomposes a non-negative data matrix X (of dimensions *m × n*) into the product of two lower-rank non-negative matrices: W (of dimensions *m × r*) and H (of dimensions *r × n*), such that the product W × H does not exceed X at any element (ie *X ≥ WH*, element-wise). In this formulation, the matrix W contains the extracted metabolic sources (ie spectral basis functions), while the matrix H encodes the source contributions across voxels or samples. Unlike standard Non-negative Matrix Factorization (NMF), which minimizes reconstruction error without restriction, G-NMU introduces an underapproximation constraint, ensuring that the estimated matrix WH remains strictly less than or equal to the observed data X. This constraint makes G-NMU particularly well-suited for sparse, interpretable, and additive source separation, especially in biological applications where it is important to avoid artificial overestimation of signal components. Least Squares decomposition was applied to quantify metabolic source contributions across voxels. The resulting sources were also projected onto non-tumor areas, allowing comparison of metabolism between tumor and healthy regions.

Dimensionality reduction was performed using Uniform Manifold Approximation and Projection (UMAP)^33^ on the G-NMU source contribution matrix. UMAP constructs a fuzzy topological graph based on local k-nearest neighbor relationships and optimizes a low-dimensional embedding that preserves these structures by minimizing cross-entropy between high- and low-dimensional graphs. Unlike PCA or t-SNE, UMAP balances local continuity with global geometry, making it well-suited for visualizing both discrete clusters and continuous gradients in complex biological data.

To mitigate overfitting, a Leave-One-Out Cross-Validation (LOO-CV) scheme was implemented. In each fold, UMAP was trained excluding the held-out patient, and only the test embedding was retained. Test embeddings from all folds were then concatenated to form the final cohort-wide representation. K-means clustering was subsequently applied to tumor-only voxels to separate metabolic profiles across astrocytoma, oligodendroglioma, and GBM subtypes.

Source contributions to UMAP structure were evaluated through linear regression-based feature importance analysis, treating standardized metabolic source values as predictors and UMAP coordinates as responses. Regression coefficients were visualized in a heatmap, highlighting the relative impact of each metabolite dimension. Finally, hierarchical clustering was applied to the z-score matrix of source contributions, generating a dendrogram and heatmap to visualize the metabolic relationships among patients and between tumor subtypes.

To evaluate metabolic differences among glioma subtypes, two complementary statistical approaches were applied. The first focused on the analysis of UMAP-based embeddings and NMU-extracted metabolic sources within tumor-only voxels. The second examined metabolic deviations between tumor and matched healthy regions. Group comparisons across AC, OG, and GBM were evaluated using the Kruskal-Wallis test for global differences, Welch’s *t*-test for pairwise comparisons under unequal variances, and the Mann-Whitney *U*-test as a non-parametric alternative. Variance homogeneity was assessed with Levene’s test. Paired tests were applied specifically to tumor vs. healthy comparisons using z-score. Effect sizes were quantified with Cohen’s d. Pearson correlation analysis was used to examine interdependencies among z-differences.

Clustering performance was quantified using the Silhouette Score, Davies-Bouldin Index, and Calinski-Harabasz. Shannon entropy was computed to assess intra-group metabolic variability, with higher values indicating increased heterogeneity. A z-score matrix was generated for each case by subtracting the mean of matched healthy tissue from the tumor mean and normalizing by the healthy standard deviation. This matrix provided standardized deviations in units of standard deviation across metabolic sources. Hierarchical clustering was performed on the z-matrix using Euclidean distance and average linkage with the Unweighted Pair Group Method with Arithmetic Mean (UPGMA), a hierarchical clustering algorithm that merges clusters based on the average pairwise distance.

For classification, UMAP embeddings were combined with K-means clustering (*K* = 3) to separate AC, OG, and GBM subtypes. Binary classification of GBM vs. AC+OG and three-class separation of GBM vs. AC vs. OG were also evaluated. Classification accuracy was assessed with leave-one-out cross-validation (LOO-CV) and receiver operating characteristic (ROC) analyses performed to compute AUC values for each subtype (AC, OG, GBM).

## Results

### Metabolic Images

Representative metabolic images for each tumor type are shown in Figure 1. Mutant IDH metabolism can be identified by high 2HG and HGI levels in the AC and OG tumors, compared to low 2HG and HGI levels of wt-IDH metabolism in GBM tumors. Choline and CNR are increased in all glioma tumors.

### Spectral Decomposition and Source Identification

**Figure 2.**
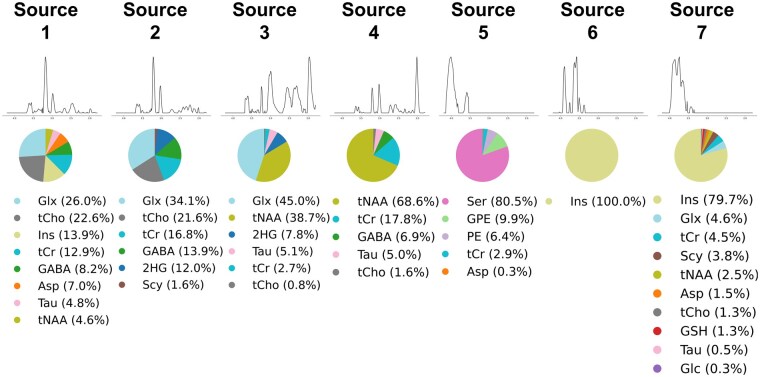
NMU-extracted spectral sources with corresponding metabolite distributions from MRSI data.

The G-NMU decomposition of the MRSI dataset revealed seven independent metabolic sources, each exhibiting unique fingerprints pertinent to glioma metabolism (Figure 2). Sources 1-2 have a metabolic profile that is more specific to glial cell metabolism. Sources 1-2 are dominated by Glx and choline, which are used as energy sources and for building cell membranes, respectively, by proliferating glioma tumor cells. In addition, Source 1 has increased myo-inositol (13.9%), which is high in glial cells metabolism, while Source 2 has an increased 2HG (12%) content, which is a marker of IDH mutation. Source 3 shows a mixed metabolic profile with a large NAA (38.7%) contribution specific to neural metabolism and a significant 2HG (7.8%) contribution specific to mutant IDH. Source 4 shows a more neural metabolic profile dominated by NAA (68.6%) and creatine (17.8%). Source 5-7 are highly enriched in metabolites that are important for glioma cell metabolism, such as serine (80.5%) in Source 5 and myo-inositol (100% and 79.7%) in Sources 6 and 7.

### Tumor Metabolic Signatures and Hierarchical Clustering

**Figure 3.**
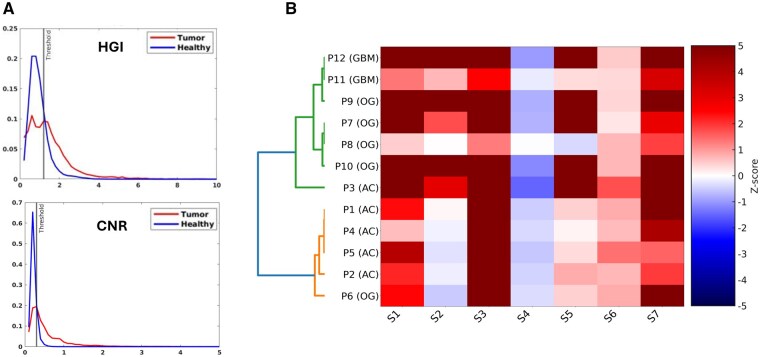
(A) HGI and CNR distribution in tumor and healthy regions and (B) Hierarchical clustering of metabolic profiles.

First we investigated metabolic alterations between the glioma tumors and the normal appearing brain. For this we used the metabolic index HGI ([2-Hydroxyglutarate + Glutamine]/[Glutamate]) to probe particularly for mutant IDH metabolism and the CNR (choline-to-NAA ratio) that probes both wild-type and mutant IDH gliomas. Tumor voxels exhibited a right-skewed HGI histogram distribution (Figure 3A) with a median of 1.55, significantly higher than the 0.91 observed in non-tumor voxels. The interquartile range (IQR) for tumor HGI values was 0.71-2.87, compared to 0.41-1.39 for non-tumor tissue, reflecting the broader metabolic heterogeneity in gliomas. The fraction of voxels exceeding the HGI > 1.5 threshold was 62% in tumors versus only 14% in non-tumor regions, demonstrating the robustness of HGI for distinguishing diseased tissue.

A similar separation was observed for CNR histograms (Figure 3A). Tumor voxels showed a median CNR of 0.58, significantly higher than the 0.27 median in non-tumor voxels. The IQR of CNR spanned 0.33-1.12 in tumors and only 0.18-0.45 in healthy tissue. The proportion of voxels exceeding the CNR > 0.4 threshold was 69% in tumors compared with 21% in non-tumor regions, underscoring the sensitivity of this index to membrane turnover and neuronal loss. Importantly, both IDH-mutant and IDH-wildtype gliomas demonstrated consistently elevated HGI and CNR values, validating the applicability of these indices across molecular subtypes.

Hierarchical clustering based on Z-scores of the G-NMU sources produced subtype-specific organization, as shown by the heatmap in Figure 3B. Inspection of the metabolite features associated with discriminating glioma subtypes indicates S1, S2, S4, S5, and S6 as the dominant sources. The inter-cluster distance between AC and OG was 0.122, compared with 0.206 between GBM and AC and 0.164 between GBM and OG, indicating stronger separation between wild-type and mutant IDH (GBM vs AC+OG) than between mIDH glioma subtypes (AC vs OG). Intra-cluster variance and entropy were 0.015 and 3.81 for GBM, 0.016 and 5.01 for OG, and 0.022 and 5.07 for AC.

### Dimensionality Reduction and Clustering Performance

**Figure 4.**
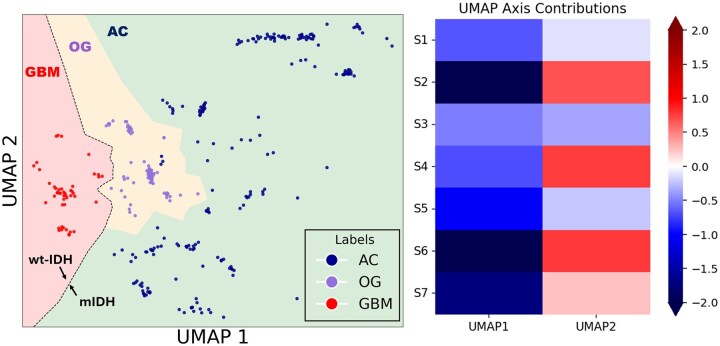
UMAP projection of metabolic profiles, clustering AC (blue), OG (purple), and GBM (red) in the left panel, and feature importance of metabolic sources in UMAP dimensions in the right panel.

To further evaluate the ability of metabolic features to classify glioma subtypes, UMAP was applied to the extracted metabolic sources. In Figure 4 (left panel), the low-dimensional representation demonstrated a clear separation between AC (red), OG (green), and GBM (blue) metabolic profiles. K-means clustering with *K* = 3 achieving a clustering accuracy of 99.65% (95% CI: 98.97%-100.00%). Sensitivity analysis indicated high recall for AC (98.15%, 95%CI: 94.55%-100.00%) with specificity reaching 100.00% (95% CI: 100.00%-100.00%) for OG and GBM. Only minimal misclassification was observed in OG cases, underscoring the robustness of the unsupervised clustering pipeline in separating glioma subtypes based on their metabolic fingerprints.

Cluster quality indices supported the robustness of this classification with Silhouette Score = 0.5062, Davies–Bouldin Index = 0.7510, and Calinski–Harabasz Index = 475.5. Together, these metrics confirmed clear inter-cluster separation with compact within-cluster organization.

Feature importance analysis (Figure 4, right panel) revealed that S2 was the strongest driver of UMAP1 and UMAP2 separation, consistent with its statistical discrimination of wild-type versus mutant IDH glioma. Source S6 contributed similarly to S2 across both axes, aligning with its group-level significance. Sources S7, S5, and S1 contributed mostly along UMAP1, while S4 contributed on both UMAP1 and UMAP2.

### Classification Performance and Statistical Validation

**Figure 5.**
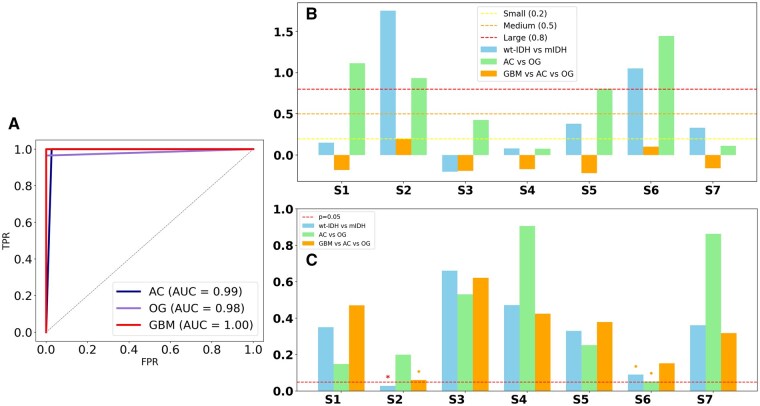
(A) ROC curve showing classification performance. (B) Effect size analysis of metabolic features. (C) T-test *P*-values for metabolite differences.

The receiver operating characteristic (ROC) analysis in ­Figure 5A showed very good classification, with an AUC of 0.99 for AC, 0.98 for OG, and a perfect separation with an AUC of 1.00 for GBM. The Kruskal–Wallis test indicated significant group differences for both UMAP1 (*P* = 3.44 × 10^−^³) and UMAP2 (*P* = 4.46 × 10^−19^). Variance analyses substantiated the heterogeneity among groups (Levene’s test for UMAP1 *P* = 2.75 × 10^−8^; UMAP2 *P* = 2.59 × 10^−^³). Effect size analysis identified large Cohen’s d values for S2 and S6 in the binary comparison of GBM versus AC+OG shown in Figure 5B. S2 had the largest effect size and statistical significance (Cohen’s *d* = 1.76, *P* = .028), whereas S6 had the next largest effect size (Cohen’s *d* = 1.05, *P* = .090). All other sources were not significant. In the comparison of three groups (GBM vs AC vs OG), S2 neared significance (*P* = .061). Pairwise Welch *t*-tests indicated that S2 was significantly higher in AC compared to GBM (*P *= .041, Cohen’s *d* = 3.55) and in OG compared to GBM (*P* = .042, Cohen’s *d* = 1.66), while no significant differences were found between AC and OG. These results underscore S2 as a reliable differentiator of wt-IDH from mIDH gliomas, while the separation of AC and OG cannot be solely attributed to univariate tests, instead indicating multivariate influences from multiple metabolic sources.

## Discussion

The preoperative classification and characterization of gliomas remain a significant challenge in neuro-oncology, which may be helped by imaging biomarkers that can capture the metabolic complexity of these tumors in a clinically meaningful manner. In this study, we applied whole-brain 3D MRSI combined with global non-negative matrix underapproximation (G-NMU), hierarchical clustering, and unsupervised machine learning to disentangle the metabolic fingerprints of astrocytoma (AC), oligodendroglioma (OG), and glioblastoma (GBM). Our results demonstrate that NMU-derived spectral sources capture biologically interpretable metabolic features that achieve near-perfect unsupervised classification of glioma subtypes. These findings provide strong support for the role of spectroscopic imaging and decomposition methods in noninvasive glioma classification. We build here on our prior research on high-resolution MRSI, which has addressed the inherent limitations of standard MRSI, particularly in terms of spatial resolution and brain coverage.^34^^,^^35^ By employing the whole-brain super-resolution metabolic mapping approach,^34^^,^^35^ this study investigates the impact of tumor heterogeneity on metabolic alterations and glioma classification. Compared to metabolic profiling studies of biopsies, our imaging data probe the entire tumor volume, capturing spatial metabolic heterogeneity.

### Metabolic Profiling and Spectral Decomposition for Glioma Classification

The application of spectral decomposition using G-NMU in this study enabled the extraction of seven distinct metabolic sources, capturing tumor-specific spectral signatures.

Spectral decomposition revealed metabolic features that are particularly enriched in different cell types. Sources 1-2 have a metabolic profile that is more specific to glial cell metabolism with major contributions from Glx, choline, myo-inositol, and 2HG. Source 3 shows a mixed glial-neuronal metabolic profile with a large NAA contribution specific to neural metabolism and a significant 2HG contribution specific to mutant IDH. Source 4 shows a more neural metabolic profile dominated by NAA and creatine. Source 5-7 are highly enriched in metabolites that are important for glioma cell metabolism,^36–38^ such as serine and myo-inositol.

### Unsupervised Learning for Glioma Classification

A novel contribution of our study is the application of NMU for spectroscopic data. Ungan et al^30^ were the first to apply NMU for MRSI-based brain tumor analysis, demonstrating that NMU variants, including sparse NMU and recursive NMU, outperform traditional NMF in extracting biologically interpretable spectral sources. Their findings indicated that NMU methods provide superior classification accuracy compared to C-NMF, particularly in distinguishing glioblastomas from low-grade gliomas. In contrast, our study combines NMU with whole-brain MRSI and super-resolution reconstruction and is the first to apply NMU to differentiate mIDH AC, mIDH OG and wt-IDH GBM.

Unsupervised clustering achieved near-perfect performance. UMAP and K-means clustering reached 99.65% overall accuracy, with an overall AUC of 0.9907 (Figure 5A). ROC analysis further demonstrated excellent subtype-level discrimination, with AUCs of 0.99 for AC, 0.98 for OG, and 1.00 for GBM. Recall was especially high for AC (98.15%), with OG achieving 96.5% accuracy and GBM achieving 100% accuracy. These results are in agreement with weakly supervised deep learning approaches that reduce reliance on manual annotations while maintaining high classification accuracy.^39–41^ Deep learning-based classification models, particularly convolutional neural networks (CNNs), have been extensively applied to glioma diagnosis using radiomic and histopathological data.^42^ However, these models predominantly rely on structural imaging, which lacks direct metabolic information. The application of UMAP in our study supports prior findings that low-dimensional embeddings can effectively capture tumor-specific metabolic heterogeneity.^43^

While only a subset of sources reached statistical significance in univariate tests, the multivariate classification performance was markedly higher. This apparent discrepancy reflects fundamental differences between the two approaches. Univariate tests evaluate each source independently and are constrained by sample size, variance heterogeneity, and intratumoral entropy, which can dilute statistical power even when effect sizes are large. In contrast, the multivariate framework integrates contributions from all sources simultaneously, capturing complementary and interacting patterns that no single metabolite or source can explain in isolation. As expected for biologically ­heterogeneous tumors such as gliomas, subtype separation arises from composite metabolic fingerprints rather than individual metabolites. Therefore, the strong clustering and ­classification results highlight the added value of multivariate decomposition in revealing clinically relevant subtype-specific metabolic profiles compared to univariate statistics.

### Comparison with Multimodal Imaging Approaches

Differentiating mIDH AC from OG remains a critical unmet need. While most contemporary studies rely on structural or perfusion MRI, radiomics, or histopathology-based deep learning, our metabolically driven approach achieved 99.65% overall accuracy, AUC= 0.9907, and per-subtype AUCs of 0.99 (AC), 0.98 (OG) and 1.00 (GBM). For instance, Zerweck et al^44^ used DCE-MRI and DKI in a 28-patient mIDH cohort (17 AC, 11 OG), reporting AUC = 0.93, sensitivity = 81%, and specificity = 100% for subtype classification based on MK, Ve, and Kep, with performance strongly driven by perfusion heterogeneity in peritumoral regions. Similarly, Mateo-Nouel et al employed 11C-MET PET in 85 gliomas to distinguish 1p/19q status, reporting SUVmax = 2.2 in OG vs. 3.7 in non-codeleted tumors, and AUC = 0.92, highlighting perfusion as a strong, though tracer dependent, metabolic proxy.^45^ Our results align biologically with these findings but stand apart in methodology: we apply a contrast-free, non-invasive, and unsupervised model based on intrinsic tumor metabolic structure, without requiring segmentation or prior knowledge of 1p/19q status. While Patel et al showed that the T2-FLAIR mismatch sign is 100% specific for astrocytomas and achieved 86.3% accuracy, this marker is visual, qualitative, and unreliable in many real-world scans.^46^ The addition of radiomics, as done by Kihira et al (FLAIR + XGBoost), improved classification to AUC = 0.89, accuracy = 88.8%, but still depends on tumor segmentation and hand-crafted imaging features.^47^

Several MRI-based deep learning studies have reported high but variable accuracies for separating IDH mutations and 1p/19q status from wild-type IDH.^48,49^ Choie et al^50^ applied CNNs and RNNs to multimodal MRI in 463 patients, achieving 95% accuracy for IDH and 89% for 1p/19q. Li et al^51^ combined CNNs with radiomics in 151 patients, reporting 96.2% accuracy, while Ge et al^52^ and Liang et al^53^ achieved 88.8% and 85.7% accuracy, respectively, using attention-based CNNs and DenseNet models. Larger multicenter studies, including Decuyper et al^54^ (466 patients) and van der Voort et al^55^ (1748 patients), reported IDH accuracies of 86.2% and 90%. Other hybrid CNN–radiomics approaches achieved more modest performance, ranging from 69% to 92% across cohorts.^56–61^

### Clustering

While OGs show somewhat lower but still elevated heterogeneity in comparison to GBM, observed differences in intra-cluster variance indicate that ACs are metabolically heterogeneous. This could be the result of metabolic ­reprogramming, 1p/19q codeletion, or microenvironmental adaptation. Crucially, UMAP embeddings showed distinct, non-overlapping isolation of the AC, OG, and GBM subtypes, highlighting the multivariate decomposition’s resilience and demonstrating that subtype differences are caused by composite metabolic fingerprints rather than common pathways. 3D MRSI examines the entire tumor volume, providing thorough metabolic signatures without subtype overlap, in contrast to focal biopsies that only take a few tumor samples.

## Limitations and future directions

The relatively small sample size (*n* = 12 treatment-naïve glioma patients) is still a limitation that could affect the findings’ generalizability, even though this study shows almost perfect classification accuracy. A larger cohort would improve the robustness of statistical comparisons and subgroup analysis, even though the dataset was meticulously selected for spectral quality, tumor burden, and molecular completeness. However, small sample sizes are typical in neuro-oncology compared to other cancers because of lower incidence and more difficult access to tissue. Several previous studies have shown that modest datasets can be used to develop accurate and generalizable machine learning models, especially when domain-guided regularization,^61^ spectral decomposition, or unsupervised learning are used. For example, using little data, deep learning frameworks have demonstrated excellent performance in applications related to radiomics, lung cancer, and neuroimaging.^62–65^ In the context of neuro-oncology, Cho et al systematically reviewed machine learning studies for brain metastasis detection and found that high performance was often achieved even with limited datasets.^66^ Moreover, it is important to note that unsupervised methods such as UMAP and K-means typically perform better when applied to larger datasets, as their underlying algorithms rely on estimating local relationships between data points. In small sample settings, neighborhood structures are harder to estimate reliably, and clustering becomes more sensitive to noise or outliers. Therefore, the fact that our model achieves clear and stable subtype separation using only twelve glioma patients indicates that the extracted metabolic features reflect strong intrinsic biological structure. This reinforces the robustness of the spectral fingerprints derived from NMU and suggests that increasing the dataset size in future studies would further enhance clustering precision and generalizability.

Additionally, while UMAP and K-means clustering ­provided effective metabolic differentiation, alternative deep learning architectures, such as graph-based clustering and attention-based neural networks, may offer further improvements in classification performance.^39^ Recent work has demonstrated that deep representation learning techniques can capture complex tumor phenotypes beyond conventional clustering methods, suggesting that these approaches could be integrated into future metabolic imaging studies. In addition, although our study was not designed to assess treatment response, the extracted spectral sources, especially those enriched in choline, NAA, and myo-inositol, have been previously associated with therapy outcomes. This raises the possibility of using source contribution changes over time (eg pre- and post-treatment) as non-invasive biomarkers for therapeutic monitoring in future longitudinal studies.

## Conclusion

This study demonstrates that NMU-based spectral decomposition of whole-brain 3D MRSI can uncover distinct metabolic fingerprints of gliomas. By integrating G-NMU with UMAP and K-means clustering, we achieved near-perfect classification in two critical tasks: distinguishing mIDH astrocytoma from mIDH oligodendroglioma and separating wt-IDH GBM from mIDH gliomas.

The extracted metabolic fingerprints revealed composite neuronal–glial patterns and selective drivers such as serine and inositol, which underpinned subtype separation.

Classification accuracy reached 99.65% (AUC = 0.9907), underscoring the robustness of metabolic fingerprints as non-invasive biomarkers.

Together, these findings position metabolic fingerprints derived from NMU as a powerful framework for precision neuro-oncology, offering a scalable pathway toward non-invasive glioma classification and future therapy monitoring.

## Data Availability

Data are available from authors based on reasonable request and with signed data shared agreement between institutions.
